# Pregnancy loss following miscarriage and termination of pregnancy for medical reasons during the COVID-19 pandemic: a thematic analysis of women’s experiences of healthcare on the island of Ireland

**DOI:** 10.1186/s12884-023-05839-4

**Published:** 2023-07-21

**Authors:** Suzanne Heaney, Martina Galeotti, Áine Aventin

**Affiliations:** School of Nursing and Midwifery, Queen’s University Belfast, Belfast, UK

**Keywords:** Pregnancy loss, Miscarriage, Termination of pregnancy, Abortion, Fetal anomaly, COVID-19, Experiences, Qualitative

## Abstract

**Background:**

Losing a baby during pregnancy can be a devastating experience for expectant parents. Many report dedicated, compassionate healthcare provision as a facilitator of positive mental health outcomes, however, healthcare services have been severely impacted during the COVID-19 pandemic.

**Aim:**

To explore women’s experiences of healthcare service provision for miscarriage and termination of pregnancy for medical reasons (TFMR) on the island of Ireland during the COVID-19 pandemic.

**Methods:**

Findings combine data from elements of two separate studies. Study 1 used a mixed methods approach with women who experienced miscarriage and attended a hospital in Northern Ireland. Study 2 was qualitative and examined experiences of TFMR in Northern Ireland and Ireland. Data analysed for this paper includes open-ended responses from 145 women to one survey question from Study 1, and semi-structured interview data with 12 women from Study 2. Data were analysed separately using Thematic Analysis and combined for presentation in this paper.

**Results:**

Combined analysis of results indicated three themes, (1) Lonely and anxiety-provoking experiences; (2) Waiting for inadequate healthcare; and (3) The comfort of compassionate healthcare professionals.

**Conclusions:**

Women’s experiences of healthcare provision were negatively impacted by COVID-19, with the exclusion of their partner in hospital, and delayed services highlighted as particularly distressing. Limited in-person interactions with health professionals appeared to compound difficulties. The lived experience of service users will be helpful in developing policies, guidelines, and training that balance both the need to minimise the risk of infection spread, with the emotional, psychological, and physical needs and wishes of parents. Further research is needed to explore the long-term impact of pregnancy loss during a pandemic on both parents and health professionals delivering care.

**Supplementary Information:**

The online version contains supplementary material available at 10.1186/s12884-023-05839-4.

## Introduction

The loss of a baby during pregnancy is an emotional and distressing event for expectant parents [1]. Miscarriage, the most common form of pregnancy loss, is classified in many countries as the spontaneous loss of a pregnancy before the 24th week of gestation [2]. It has been estimated that 10–20% of clinical pregnancies end in miscarriage annually in the United Kingdom (UK)[3] with similar figures reported globally [4]. The experience of miscarriage is reported as a traumatic event for many parents and research has indicated that, for many women, it can result in the development of long- and short- term psychological difficulties such as depression, anxiety, and post-traumatic stress disorder [3, 5].

Another type of loss during pregnancy results from termination of pregnancy for medical reasons (TFMR), also referred to as termination of pregnancy for fetal anomaly (TOPFA). TFMR generally follows diagnosis, during pregnancy, of congenital anomalies involving severe structural and/or functional abnormalities. Legal differences across the globe regarding the definition and availability of TFMR have resulted in unreliable statistics and limited data for comparative purposes internationally [6, 7]. Nonetheless, European figures suggest a TFMR prevalence rate of 4.6 per 1,000 births [8]. In the UK, over 70% of congenital anomalies are detected during pregnancy and, of those, around 37% will result in TFMR [9]. An increasing body of literature acknowledges the complex grief experience of parents experiencing TFMR [10–12], many of whom report disenfranchised grief, limited support, and feelings of stigmatisation and shame due to negative societal attitudes and legal and clinical constraints in respect of termination of pregnancy [1, 13, 14].

In March 2020, the World Health Organisation declared a worldwide pandemic caused by the SARS-COV-2 Virus (COVID-19) [15]. This has severely affected healthcare provision, resulting in unprecedented pressure on both healthcare systems and healthcare professionals [16]. In many countries, many important features of maternity care that had previously supported women’s emotional and psychological health were reduced or removed to ensure continued provision of acute clinical care [17–19]. In the UK, most hospital sites reported a reduction in scheduled antenatal appointments (by 70%), postnatal appointments (by 56%) [20], and social support and community healthcare services [21, 22]. Burgeoning research relating to COVID-19 and maternity care has highlighted the impact of the disease on maternal and neonatal morbidity and mortality [23–25] and its psychological impact on health professionals [26–28]. However, less is known about its impact on parents experiencing pregnancy loss [20, 29].

This paper adds to the literature by reporting parents’ experiences of healthcare service provision on the island of Ireland. The paper combines findings from two separate studies relating to women who have experienced miscarriage (up to 24 weeks) and TFMR. While we recognise that there are differences between these experiences of loss, our findings from the broader studies indicated similar experiences in negotiating the healthcare system during the COVID-19 pandemic. There is a substantial body of research which confirms that the loss of a baby during pregnancy can be associated with long-term grief reactions regardless of gestational age or whether the pregnancy was terminated spontaneously or intentionally [30, 31]. It is argued that the degree of parental investment in and attachment to the baby, rather than form of loss is the most relevant predictor of grief and experiences of loss [32–35]. With COVID-19 and similar viruses predicted to be a persistent part of the future [36–38], understanding parents’ experiences of pregnancy loss, grief, and recovery will help to design and develop healthcare systems capable of providing optimal care.

## Aim

The aim of this paper is to explore women’s experiences of healthcare service provision for miscarriage and termination of pregnancy for medical reasons (TFMR) during the COVID-19 pandemic.

## Methods

This paper combines datasets from two separate studies conducted during the COVID-19 pandemic 2020/21. Study 1 was conducted in Northern Ireland (NI) and Study 2 included participants from NI and the Republic of Ireland (IE). Methods and findings are reported using the Consolidated Criteria for Reporting Qualitative Research (COREQ) Guidelines [39]. Both studies adopt an approach aligned with the interpretivist-constructivist paradigm implying an ontological and epistemological position in which truth is viewed as being subjective and culturally based, and an individual’s experiences help them to interpret the world. Research based on this paradigm focuses on exploration of the way people interpret and make sense of their experiences in the world in which they live [40].

### Study design

Study 1 was a mixed methods needs assessment focusing on how to better support the emotional needs of women who experience miscarriage in hospital settings in NI. It involved an online survey of women who had experienced miscarriage and attended hospital settings in NI within the previous 5 years. The survey was developed by the research team in consultation with an Advisory Group and contained The Reversed Impact of Miscarriage Scale [41] and questions adapted from a study on maternity care previously conducted in Ireland [42]. Study 2 was a qualitative study carried out in NI and IE to explore the healthcare needs and experiences of female and male parents who have had a TFMR. For the purposes of this paper, only data from the studies related to parent’s experiences of care during COVID-19 are reported.

### Settings

This study took place on the island of Ireland, which is the third largest island in Europe. Ireland is comprised of two jurisdictions, IE, which is part of the European Union, and NI, which is a part of the UK. IE constitutes approximately five-sixths of the island, while NI covers the remainder. The approximate population of IE is 5 million with 1.9 million in NI [43]. Although Northern Ireland and Ireland are independent states located on the same island, they are culturally and legally similar, with comparable maternal and child health service provision. Further, private health insurance and cross-border working on the island means that some women travel across the border to access health care.

TFMR became legal in IE following a referendum in May 2018 and abortion services commenced in January 2019 [39]. In NI, abortion was decriminalised in October 2019 following the Northern Ireland (Executive Formation etc.) Act 2019 [44, 45]. At the time of writing, abortion services, as outlined in the NI abortion regulations, have not yet been commissioned by the Department of Health Northern Ireland. However, a limited service for early medical abortion to those under 10 weeks gestation is provided in some areas [41, 45].

### Participants

The study combines data from a total of 157 participants, 145 from Study 1 and 12 from Study 2. A purposive sampling strategy was used for both studies, and parents were recruited through special interest groups, organisations online, and social media platforms. For Study 2, a short animation recruitment video (https://www.youtube.com/watch?v=dVPt4gLz9iE) was co-designed with the study’s advisory group. All participants were self-selecting and written informed consent was obtained prior to data collection.

Most of the participants included in Study 1 (n = 145), were aged between 31 and 39 years old (57%), only had one miscarriage (63%), and lived with their partner and other children (57%). More detailed information can be found in Table 1.

**Table 1:** Participant Characteristics Study 1

Demographic Information	No. of Participants	Percentage
**Age**		
16–25	16	11%
26–30	36	25%
31–39	83	57%
40–49	10	7%
Total	145	100%
**Number of miscarriages**		
1	91	63%
2	31	21%
3	12	8%
4+	11	8%
Total	145	100%
**Number of weeks when miscarriage occurred**		
Between 4 and 6 weeks	17	12%
Between 7 and 12 weeks	108	75%
Between 13 and 16 weeks	7	5%
Between 17 and 20 weeks	7	5%
Between 21 and 24 weeks	5	3%
Don’t know	1	1%
Total	145	100%
**Living arrangements**		
Alone	4	3%
With partner and other children	83	57%
With partner	58	40%
Total	145	100%
**Education**		
Higher Degree	16	11%
BSc Degree	36	25%
Higher/A-levels	83	57%
Standard grade/GCSE	10	7%
Total	145	100%

Study 2 included the experiences of 12 women. Participants were aged 18 years and over living on the island of Ireland and had had their TFMR in 2020 or 2021. The pregnancy gestation when parents had a TFMR ranged from 13 weeks to 27 weeks, with the majority (n = 7) taking place between 20 and 24 weeks gestation. This reflects the fact that most parents find out about a congenital anomaly at the routine fetal anomaly screening ultrasound scan, carried out in mid-pregnancy (18–22 weeks gestation) [42, 43, 46–48]. More information can be found in Table 2.

**Table 2:** Participant Characteristics Study 2

Characteristic	No. of Participants
**Parents**	
Women	12
**Jurisdiction of Experience**	
Republic of Ireland	7
Northern Ireland	5
**Year of TFMR**	
2020	7
2021	5
**Gestation at TFMR**	
<13 weeks	0
13–18 weeks	3
18–20 weeks	0
20–22 weeks	2
22–24 weeks	5
24–28 weeks	2
>28 weeks	0
**Total**	12

### Data collection

For Study 1, Qualtrics software (Qualtrics, 2022) was used to design and administer a survey to explore women’s perceived emotional support needs in hospital settings when they attended the facility due to miscarriage. A link to the survey was distributed by MG using social media platforms such as Facebook and Twitter between February and April 2021. The survey, used as part of a larger doctoral study contained an open-ended question for those women who had their miscarriage during the COVID-19 pandemic. They were asked to describe how COVID-19 had impacted on their experience of miscarriage in hospital settings.

For Study 2, two methods of data collection were offered to all participants, semi-structured interview and written narrative account. The interview schedule consisted of open-ended questions about healthcare experiences, with specific attention to engagement with healthcare professionals and care processes. Narrative account was used as a means of complementing interview data and to enable participants to share their stories in more detail [49]. It was also offered to any parent who declined or was unable to take part in an interview. Participants choosing to submit a narrative account were asked to provide a written account of any length, which detailed their experiences. Data collection took place between November 2020 and August 2021. Eleven interviews were carried out by SH on a video platform (Microsoft Teams) and one was face-to-face, conducted in line with Government and University COVID-19 Guidelines. All interviews were recorded using an audio recording device and lasted between 48 min and 3.47 h (average time – 110 min).

### Data analysis

#### Study 1

Anonymised open-ended responses from the survey were downloaded from Qualtrics into NVivo 12 for analysis. Open ended questions were thematically analysed using the Braun and Clarke framework (2006), where initial codes were identified and subsequentially organised in themes [50–53]. To ensure confidentiality, participants were allocated a code. Women were assigned a unique identifier code, for example ‘P01-miscarriage’ to represent Participant 1, Study 1.

#### Study 2

Audio recordings of interviews were transcribed verbatim and imported to NVivo 12. To preserve confidentiality, participants were allocated a code. Women were assigned a unique identifier code, for example, ‘P01- TFMR’ to represent Participant 1, Study 2. Thematic analysis was conducted on the open-ended questions following the Braun and Clarke framework. Initial codes were identified and subsequentially organised in themes [50]. To help ensure trustworthiness of the data, participants were provided with a summary of their interview for member checking [54]. Of those who responded, all were satisfied that the summary was accurate. While member checking is used extensively in qualitative research, the myriad of formats or approaches it can entail, leaves it open to criticism with few researchers reporting in detail the process they used, and some argue it can often be a tokenistic gesture [55–57]. In this study a summary in the form of bullet points was provided to parents. This abbreviated approach was agreed with the study advisory group to demonstrate that the researcher had understood participants correctly while not over-burdening them.

Data were analysed separately for each study by MG (Study 1) and SH (Study 2). Interview transcripts, text-based survey responses, and written narrative accounts were read several times to immerse the researchers in the data, and open inductive coding was carried out. Following initial thematic analysis, the co-authors met to critically discuss coding, compare findings, discuss theme labels and definitions, and agree representative participant quotes to illustrate themes. For Study 2 when data saturation was reached transcripts were compared to ensure that the most representative quotations were selected and presented in the results Sect. [58].

Data collection materials were developed by the PhD researchers [SH, MG] in close consultation with their primary supervisor [ÁA]. SH and MG conducted the analysis, with coding and final analysis checked by ÁA. All authors are trained to MSc level in qualitative and quantitative research methods. ÁA also has a PhD and almost 20 years’ experience in the conduct of public health research.

### Findings

The majority of participants in both studies perceived COVID-19 as having a negative impact on their healthcare experiences. The analysis resulted in three main themes: (1) lonely and anxiety provoking experiences; (2) waiting for inadequate healthcare; and (3) the comfort of compassionate healthcare professionals.

#### Theme 1: lonely and anxiety-provoking experiences

There was unanimity among women in both studies that losing their baby was a challenging and emotional experience. COVID-19 appeared to compound what was already a painful experience with heightened feelings of anxiety and loneliness.

“COVID-19 has made it a very lonely experience. Scans to confirm baby had passed was alone, appointments, hospital stay all alone. Was awful and certainly affected me worse that any of my other losses” (P15-miscarriage).

“The hardest thing, due to COVID, was that I had to be on my own for all of this” (P16-TFMR).

Absence of partner presence at appointments and in hospital settings was mentioned by most participants. When partners were able to attend appointments, scans, investigations, or meetings despite COVID-19 restrictions, both parents expressed gratitude and valued its importance:

“All this is in COVID and (partner) was allowed to come to all of those appointments there was no question about it. You know they were like ‘absolutely’; it was never a ‘oh I’ll need to check that’, or ‘can you phone on the day’ it was like ‘no, you need your husband’, it was not even a question, which was massive” (P11-TFMR).

When partners were excluded from any part of the process, it resulted in distress and frustration.

“We should have been there as a couple” (P20, TFMR).

“Having to attend hospital alone while haemorrhaging was awful, I felt so unwell and if my partner could have accompanied me it would have been a lot less frightening” (P84, miscarriage).

“The pandemic made things 100 times worse as I had to attend many scans and be told I was having a miscarriage without the support of my partner” (P66, miscarriage).

“Even when they were discussing my options, I had to make all the decisions on my own. Makes the whole experience harder to go through” (P80, miscarriage).

Similarly, some women reported that it was upsetting to have to deliver the news to their partners.

“Whilst I tried to take everything in that I was told, it was very difficult, and it was even more difficult to try and relay the info back to my partner” (P115, miscarriage).

“The news was bad enough but the thought of arriving home and having to tell the news again to my partner caused untold anxiety. How do you say the words? How do I tell him our baby may die?” (P20, TFMR).

The possible implications of contracting the virus and mandatory COVID-19 tests were also anxiety-provoking, causing some to worry about the impact a positive result would have on their access to care, care options, and processes:

“I drove to the COVID test like a woman possessed. I hadn’t slept in days wondering if I would be the one who tested positive with no symptoms. What if I couldn’t fly [to England for the termination] and had to isolate for 14 days – it would be too late. What if he [partner] tested positive and couldn’t come with me?” (P20, TFMR).

“Never cared if I got COVID but was worried in case it harmed the unborn baby” (P140, miscarriage).

#### Theme 2: waiting for inadequate care

Participants commented on how COVID-19 impacted negatively on the provision, accessibility, and efficiency of the services they were navigating, and this was identified as a major contributor in parent dissatisfaction. Issues such as delayed investigative procedures and test results due to COVID-19 for participants were a major source of frustration.

“The test might take two weeks, but with COVID it could take up to eight weeks, he [doctor] said” (P20, TFMR)

“Due to COVID there was a postage delay with all the [testing] kits and there wasn’t enough for my appointment” (P16, TFMR).

Similarly, some women indicated that COVID-19 had an impact on hospital appointments resulting in delayed, prolonged, or cancelled treatment.

“[COVID-19] resulted in extremely traumatic experience where I was sent home to let ‘nature’ take its course. Then tried medical management which was unsuccessful. Four weeks later I had surgery. Longest 4 weeks of my entire life” (P08, miscarriage).

“I [was] […] still so dizzy at this stage […] still bleeding quite heavily. […] And I was like, can you disconnect me from this drip, because I need to go to the toilet? And she [nurse] was like, I’m not actually allowed to come into your room because your COVID test isn’t back yet” (P18, TFMR).

Some women perceived that their appointments with health professionals were rushed, and that the professionals did not want them there:

“It was a very rushed appointment, I was not given any support and it was all very abrupt. I was not given any guidance or support materials and was rushed out [of] the appointment with no understanding of what was going on or what would have happened next” (P90, miscarriage).

Some participants explained the difficulties of accessing services and booking face-to-face assessment appointments:

“I was told over the phone I was having a miscarriage and I was never medically examined to check it was not an ectopic pregnancy or a complete miscarriage” (P59, miscarriage).

Some respondents reported that they were either re-directed home after attending hospital or asked not to come into hospital to be assessed and this was perceived as distressing and traumatic:

“While I felt I was miscarrying for weeks, I was told to remain at home prolonging the uncertainty of “was I or wasn’t I?” Left me afraid to go to work or doing anything for fear of bringing on a miscarriage” (P02, miscarriage).

Some women explained that they were unable to avail of their preferred treatment option because some procedures were suspended during the pandemic:

“To not offer women surgical option is barbaric. To make me repeatedly take tablets and have to beg for surgery and eventually when I got it to tell me how lucky I was, not acceptable” (P124, miscarriage).

“My miscarriage was horrific, […] I was left for weeks on end waiting for a miscarriage to happen that was never going to happen on its own. I was denied an MVA [manual vacuum aspiration] in a timely manner due to a regional decision taken to stop surgeries” (P145, miscarriage).

One participant commented on the availability of services being limited due to the impact of COVID-19 on training health professionals.

“There’s this sort of thing where, oh well nobody has been trained because of COVID-19. And that makes you angry” (P21, TFMR).

For patients who needed to travel from Ireland to Great Britain (GB) to access care, COVID-19 impacted significantly on that experience. Several commented on the fear of the high COVID-19 case numbers in GB at the time (P18, TFMR). One major obstacle was accessibility:

“With the pandemic traveling is a lot harder, a lot of hospitals aren’t taking people anymore. It’s really hard to get an appointment anywhere” (P07, TFMR).

“The lady talked me through different places to contact in the UK as hospitals were no longer taking women from abroad […] The consultant called the next day to say she did all she could but they won’t take anyone in Liverpool from outside the UK and no hospital was” (P20, TFMR).

#### Theme 3 the comfort of compassionate healthcare professionals

Participants in both studies greatly valued when healthcare professionals provided compassionate and empathetic care during their pregnancy loss experience:

“Whilst it was hard going through it during a pandemic, the midwives who supported us made the experience a little less traumatic. Those midwives are the most amazing women, myself and my partner are eternally grateful for their support throughout the single most tragic and awful experience of our lives” (P74, miscarriage).

Similarly, the human touch from healthcare professionals was also greatly appreciated by participants.

“She just hugged me and she’s like I know I’m not supposed to but I just I have to hug you and like she was just lovely” (P16, TFMR).

“She hugged us and said goodbye in COVID times” (PM03, TFMR).

Conversely, one participant noted a perceived lack of empathy and kindness from healthcare professionals impacted negatively on participants’ experience:

“She [nurse] came in then, eventually, and I was really upset and I just said to her, ‘I’m so scared’, I said, ‘I don’t want to be on my own’. And she was just so harsh, and she said, ‘well you have to, you’ve no choice. You went to England and COVID’s really bad over there in the UK, so you have no choice, so you have to’” (P18, TFMR).

## Discussion

This study highlights parents’ experiences of healthcare provision for miscarriage and TFMR during the COVID-19 pandemic. Findings of this study highlight the similarity of experiences reported by women experiencing two very different types of loss, across different jurisdictions with different healthcare systems. To our knowledge, this is the first study to include data on participants’ experiences of loss due to different pregnancy loss conditions from across the island of Ireland. Parents’ experiences were mainly negative, often perceived as lonely and characterised by delayed or unavailable treatments, cancelled hospital appointments, reduced services and a lack of support and empathy from healthcare professionals. Positive experiences were also reported, centered on examples of compassionate care from healthcare professionals. These findings reflect those of other international studies conducted on maternity services during COVID-19 [20, 29, 59–61].

Our findings indicate the primary importance of support from partners and compassionate healthcare professionals during the process of grieving pregnancy loss, support that was profoundly interrupted during the pandemic. These findings are in line with other research, conducted both during the COVID-19 pandemic[59, 60, 62, 63] and prior to it[64–66], reporting the necessity of involving partners in maternity care. Our findings highlighted the desire and need for both mothers and fathers to be involved in the loss experience, with participants finding the exclusion of partners due to COVID-19, to negatively compound their already ‘traumatic’ experience. While historically men were not involved in the pregnancy or birth experience, a shift in the pregnancy and childbirth continuum sees modern maternity services expecting the presence of the father/partner [67]. This attitude was echoed in this study, with participants seeing the parent couple as a unit that should be treated as one entity. Recently published UK bereavement guidelines for pregnancy loss in COVID-19 support and acknowledge the importance of having partners present during this experience [68]. However, this guideline appears to have been inconsistently applied in practice during the pandemic. Moving forward there is a need for clear and accountable policies regarding the exclusion of partners with consideration of the psychological impact on both parents. Further research could explore the long-term impacts on both parents of partner exclusion during pregnancy loss.

Relatedly, the findings of this study reflect the important role that healthcare professionals can play in promoting positive mental health outcomes for women and how this was gravely impacted by the restrictions imposed during the pandemic. While best practice guidelines and theories on breaking bad news in healthcare settings advise a face-to-face conversation with the presence of a relative [69–73], findings from our study suggest that practice in relation to this was inconsistent and disappointing for many. Consistent with other research [69], the potential for negative impacts on the emotional wellbeing of parents due to a lack of such supportive conversations was reported. It is possible that this situation was a result of healthcare professionals not having adequate training and guidance to address these difficult conversations remotely, a finding echoed by other research [74]. Participants also reported a disparity between parents’ desired level of information and what was provided in practice. This was particularly the case in relation to a reported lack of information provided on available aftercare, and specific details on what to expect for those having a miscarriage at home. It is unclear whether this is an entirely COVID-19 specific issue or reflective of practice outside of this pandemic. However, literature regarding pregnancy loss highlights the importance of good communication and appropriate, balanced, and multi-formatted information [75–78]. Research from the perspective of both service users and healthcare professionals would be useful to inform best practice regarding communication and information provision during a pandemic and beyond. Further, attention needs to be given to the development of evidence-informed guidelines and policies regarding compassionate care and delivering sensitive news remotely. In addition, prompt action is needed to enhance health professionals’ competence and skills in delivering care and information through non-face-to-face methods.

Another key issue raised by the findings of this study relates to the disruptions caused by COVID-19 on the timeliness and quality of services provided. Echoing findings from other research [64], we found increased anxiety, anger, and distress resulting from restrictions regarding physical attendance at hospital and limited choice in relation to miscarriage management. Such restrictions are directly opposed to guidelines from those such as the UK National Institute for Health and Care Excellence (NICE), which advocates the importance of women’s preference in choosing the treatment of miscarriage (expectant, medical or surgical) [2]. While the long-term physical and emotional impacts of the lack of choice on parents are unknown, ‘choice’ within healthcare is a fundamental element of patient satisfaction and effective and high-quality care, supported widely by literature and best practice guidelines [79–83]. Further research is needed to examine the lived experience of parents regarding service provision during a pandemic to inform future policies that balance the need to minimise the risk of virus transmission with the emotional, psychological and physical needs and wishes of service users.

### Strengths & limitations

This is a timely study, reporting in-depth the healthcare experience of parents who have lost a baby during pregnancy in the course of the current COVID-19 pandemic. The response to recruitment was extremely positive in both studies, suggesting that there is a need for further examination of these issues. This study provides the first examination of the impact of COVID-19 restrictions on experiences of healthcare provision from the perspective of women across the island of Ireland experiencing loss due to different conditions. The findings are clear that, although experiences and decision-making relating to miscarriage and TFMR are different, the need for support and responsive services are paramount.

The findings are limited by the type of loss, participants, and study setting. In addition to the recommendations noted above, further research could explore this phenomenon further from these other perspectives, by examining, for example, pregnancy loss following ectopic pregnancy and among women in other parts of the UK. Exploring this experience within the context of COVID-19 was not the primary aim of the two respective studies and a study entirely focused on this context could add further depth to the findings. The sample in both studies is limited to an ethnically homogenous population from the island of Ireland. Future research in other contexts could explore the pregnancy loss experiences of parents from other ethnic communities. Further, the authors would like to note that data from an open-ended question in the survey might not be as rich as data from in-depth interviews. However, the authors assumed that responses to the open-ended question reflected what was important to women and therefore, they were thematically analysed [52, 53].

## Conclusion

This paper, through the lens of bereaved parents, has provided insight into the pregnancy loss healthcare experience during the COVID-19 pandemic. Losing a baby at any stage of pregnancy is an extremely distressing experience and parents reported that the limitations and restrictions of partner presence in maternity settings compounded this. As COVID-19 increasingly appears to be a challenge that we must grapple with in the longer term, it is imperative that healthcare professionals establish how to provide both an effective service and compassionate care to families losing a baby that minimises the risk of infection and spread of COVID-19 but also ensures a supportive experience.

## Electronic supplementary material

Below is the link to the electronic supplementary material.

Supplementary Material 1

## Data Availability

Data cannot be shared publicly because of ethical restriction placed upon the data for the following reasons: data contains potentially identifying and sensitive information. Interview transcriptions might expose participants identity if shared publicly; researchers did not obtain consent from participants to share raw data publicly at the time of data collection. Point of contact for data enquiries - Corresponding author Martina Galeotti, MG,
