# Supplementary material for: Pregnancy loss following miscarriage and termination of pregnancy for medical reasons during the COVID-19 pandemic: a thematic analysis of women’s experiences of healthcare on the island of Ireland
Source: BMC Pregnancy Childbirth. 2023 Jul 21;23:529. doi: 10.1186/s12884-023-05839-4 (PMC10360341; doi:10.1186/s12884-023-05839-4)
Supplement: Supplementary file 1 — Supplementary Material 1 [file 12884_2023_5839_MOESM1_ESM.docx]

**Additional File: Illustrative quotes representative of themes**

| Theme | Quote |
| --- | --- |
| THEME 1  **Loneliness and Anxiety-Provoking Experiences** | “Finding out I was experiencing miscarriage without the support of my husband was extremely difficult on both of us” (P153, miscarriage)  “The day I attended the early pregnancy unit there was no other patients there and the waiting room was completely empty. I feel my partner should have even been allowed to sit in there to wait on me to avoid the horrendous walk into the hospital on your own, walking past mothers and fathers carrying their new born baby’s out passed you” (P143, miscarriage)  “Had the MRI. Again my partner wasn’t allowed into the hospital so I had to go in to have that done … at the next meeting then was the same, my partner had to stand outside until we got into the meeting room” (P18, TFMR)  “The hardest thing, due to COVID, was that I had to be on my own for all of this” (P16, TFMR)  “All this is in COVID and (partner) was allowed to come to all of those appointments there was no question about it. You know they were like ‘absolutely’; it was never a ‘oh I’ll need to check that’, or ‘can you phone on the day’ it was like ‘no, you need your husband’, it was not even a question which was massive” (P11, TFMR)    “We should have been there as a couple” (P20, TFMR)    “Having to attend hospital alone while haemorrhaging was awful I felt so unwell and if my husband could have accompanied me it would have been a lot less frightening” (P84, miscarriage)  “The pandemic made things 100 times worse as I had to attend many scans and be told I was having a miscarriage without the support of my partner” (P66, miscarriage)  “The news was bad enough but the thought of arriving home and having to tell the news again to my partner caused untold anxiety. How do you say the words? How do I tell him our little girl may die?” (P20, TFMR)  “Whilst I tried to take everything in that I was told, it was very difficult, and it was even more difficult to try and relay the info back to my husband” (P115, miscarriage) |
| THEME 2  Waiting for Inadequate Care | “I was unable to attend my follow up appointment at the early pregnancy clinic [….] it was cancelled due to COVID-19. I was due to attend for a scan to confirm everything had passed” (P105, miscarriage)  “I was unable to receive anaesthetic for my procedure because of COVID-19” (P48, miscarriage)  “No hospital would take us because of COVID” (P20, TFMR)  “The test might take two weeks, but with COVID it could take up to eight weeks, he said” (P20, TFMR)  “Due to COVID there was a postage delay with all the [testing] kits and there wasn't enough for my appointment the following evening” (P16, TFMR)  “While I felt I was miscarrying for weeks, I was told to remain at home prolonging the uncertainty of “was I or wasn’t I?” Left me afraid to go to work or doing anything for fear of bringing on a miscarriage” (P02, miscarriage).  “[COVID-19] resulted in extremely traumatic experience where I was sent home to let nature take its course. Then tried medical management which was unsuccessful. Four weeks later I had surgery. Longest 4 weeks of my entire life” (P08, miscarriage)  “It was a very rushed appointment, I was not given any support and it was all very abrupt. I was not given any guidance or support materials and was rushed out the appointment with no understanding of what was going on or what would have happened next” (P90, miscarriage)  “I was told over the phone I was having a miscarriage and I was never medically examined to check it was not an ectopic pregnancy or a complete miscarriage” (P59, miscarriage)  “To not offer women surgical option is barbaric. To make me repeatedly take tablets and have to beg for surgery and eventually when I got it to tell me how lucky I was, not acceptable” (P124, miscarriage)  “My miscarriage was horrific, […] I was left for weeks on end waiting for a miscarriage to happen that was never going to happen on it’s own. I was denied an MVA [manual vacuum aspiration] in a timely manner due to a regional decision taken to stop surgeries” (P145, miscarriage)  “There’s this sort of thing where, oh well nobody has been trained because of COVID-19. And that makes you angry” (P21, TFMR)  “With the pandemic traveling is a lot harder, a lot of hospitals aren't taking people anymore. It's really hard to get an appointment anywhere” (P07, TFMR)  “The lady talked me through different places to contact in the UK as hospitals were no longer taking women from abroad … The consultant called the next day to say she did all she could but they won’t take anyone in Liverpool from outside the UK and no hospital was” (PW20, S2) |
| THEME 3  **The Comfort of Compassionate Health care Professionals** | “While after my miscarriage I could not fault the staff for their sensitivity and for taking into account my previous loss - it was incredibly distressing attending scans alone, and receiving the news I had miscarried without my partner present” (P131, miscarriage)  “It was all masked and horrible, my tears and the mask were rotten” (P13, TFMR)  “I knew as I looked at her confused eyes, her face hidden by her mask. But the eyes never lie” (P20, TFMR)  “Whilst it was hard going through it during a pandemic, the midwives who supported us […] made the experience a little less traumatic. Those midwives are the most amazing women, myself and my partner are eternally grateful for their support throughout the single most tragic and awful experience of our lives” (P74, miscarriage)  “She came in then, eventually, and I was really upset and I just said to her, ‘I’m so scared’, I said, ‘I don’t want to be on my own’. And she was just so harsh, and she said, ‘well you have to, you’ve no choice. You went to England and COVID’s really bad over there in the UK, so you have no choice so you have to’” (P18, TFMR)  “She just hugged me and she's like I know I'm not supposed to but I just I have to hug you and like she was just lovely” (P16, TFMR) |
